# Assessment of awareness level about consequences of missing teeth in Qassim region, Saudi Arabia

**DOI:** 10.1371/journal.pone.0322325

**Published:** 2025-05-20

**Authors:** Rayan Saleh Al-Mutairi, Bayan Almohaimeed

**Affiliations:** 1 College of Dentistry, Qassim University, Qassim, Saudi Arabia; 2 Department of Community Dentistry and Oral Epidemiology, College of Dentistry, Qassim University, Qassim, Saudi Arabia; Far Eastern Memorial Hospital, TAIWAN

## Abstract

Missing teeth is a prevalent dental health problem that can lead to several unfavorable consequences, including dental caries, periodontal disease, bone deterioration, jaw disorders, malocclusion, and irregular teeth alignment. This Study aims to assess awareness of the consequences of missing teeth in the Qassim region, Saudi Arabia. A cross-section study was conducted, including 216 Saudi adults from Qassim, while children were excluded. Data were collected using an online questionnaire, which consisted of two parts: the first part gathered participants’ demographic data, and the second part included questions assessing their awareness level regarding teeth loss. Descriptive statistical analysis, bivariate chi-square test, and multivariate logistic regression analysis were performed using SAS OnDemand for academics. Among the 216 participants, 129 (59.7%) were females and 69 (31.9%) were between the ages of 26 and 35. The study revealed that 60.2% of participants were knowledgeable about treatment options for missing teeth. However, a lack of knowledge regarding available treatment options was significantly associated with age (P-value < .0001), education level (P-value 0.0336), and income level (P-value 0.0037). In this study, only 62.5% of participants reported being aware of the consequences of missing teeth. A significant number of the participants lacked awareness of these consequences, with the highest percentage of unawareness observed among the uneducated. Hence, educating patients about the complications of not replacing missing teeth is crucial, as it can improve their attitude toward treatment and enhance their quality of life.

## Introduction

Tooth loss is a widespread dental health problem and one of the most common dental conditions [1]. It can be a result of dental diseases such as dental caries, periodontal disease, infection, or tumors and may also be caused by trauma or failed dental treatments [2–5]. Tooth loss disturbs an individual's physical and mental health, quality of life (QoL), and overall well-being [4,5]. Furthermore, it can cause difficulty and discomfort in daily activities, such as speaking, eating, drinking, and social interaction [1,6].

Missing teeth negatively impact oral health, leading to over-eruption, rotation, or drifting of adjacent teeth, which can result in tooth loss, uneven wear, temporomandibular joint disorder (TMDs), and facial or oral midline shift (asymmetry) or collapse [6–10]. Moreover, untreated teeth drifting can cause dental caries, gingival and periodontal disease, and bone loss. Negative dental and oral consequences often arise due to the difficulty of maintaining good oral hygiene and food debris removal from narrow contact areas [11].

Various treatment options are available in the dental office for replacing missing teeth, including fixed crowns and bridges, removable dentures, and implant-supported dentures. These treatments can be performed by prosthodontics alone or by a team of multidisciplinary dentists.

The main goal of replacing missing teeth is to restore the patient’s aesthetic and function. The appropriate treatment choice depends on individual cases and sociodemographic factors such as income level and education [1,6]. Recently, dental implants have gained popularity due to their minimal risks and long-term benefits.

Awareness of the consequences of not replacing missing teeth is a crucial step toward optimum oral and dental health. Good oral hygiene practices and routine dental care are key to preventing dental and oral diseases such as dental caries and periodontal disease [12,13]. Dentists should consider their patient’s knowledge, awareness, education, and socioeconomic status when planning treatment alternatives [14]. Non-compliance with oral hygiene instructions and regular dental visits often stems from inadequate knowledge and lack of awareness, which may ultimately lead to tooth loss [15].

The necessity of this study is emphasized by the lack of data regarding the Saudi population’s awareness levels concerning the implications of tooth loss. Therefore, the goal of this study is to assess the awareness of the consequences of missing teeth among individuals in Qassim, Saudi Arabia.

## Materials and methods

A cross-sectional study was conducted among the Qassim population aged 18 and above; children were excluded. Ethical approval was obtained from the Qassim University Research Center Ethical Committee (#24-73-04). Participants provided written consent before participating in the study. Data were collected through an online electronic questionnaire consisting of two parts, comprising Eighteen closed-end questions. The first part included four questions regarding participants’ demographic data, such as age, gender, education, and income level. The second part contained fourteen questions assessing participants’ awareness of the consequences of tooth loss and prosthodontics treatment options. The questionnaire questions were adopted from a study by alshehri [16]. The language was originally in English and was translated into Arabic to ensure better understanding.

Additionally, a question was included to determine whether participants had missing teeth. If they answered yes, two follow-up questions were asked: (1) how many teeth were missing? and (2) What was the reason for teeth loss? Furthermore, if the participants answered yes to “Are you aware of the consequences of missing teeth?”, they were asked about the source of their knowledge. The questionnaire was distributed via social media. Data collection took place between February 18, 2024, and March 31, 2024. Descriptive statistical analysis, bivariate chi-square test, and multivariate logistic regression analysis were executed using SAS On Demand for Academics. Descriptive statistics (frequency and percentage) were used to quantify the variables in this study. The correlation between categorical data and outcome variables was examined using the Chi-square test. A multivariate logistic regression analysis was conducted to examine the association between participants’ demographic data, self-reported awareness level, and teeth-related issues. A statistically significant difference was considered at a P-value of <0.05.

## Results

Out of 216 participants, 129 (59.7%) were female, and 31.9% were in the 26–35 age group. The study reported no cases of illiteracy among the participants. Regarding education level, 10 participants (4.6%), had completed elementary education, 20 (9.3%) had secondary education (middle school), and 60 (27.8%) had tertiary education (high school). Additionally, the majority of participants, 111 (51.4%), had attained higher education (university level) (Table 1).

**Table 1:** participants’ Sociodemographic characteristics.

Sociodemographic characteristics	n (%)
***Age groups (years)***	
18–25	54 (25.0%)
26–35	69 (31.9%)
36–45	40 (18.5%)
46–55	41 (19.0%)
56–65	11 (5.1%)
>65	1 (0.5%)
***Gender***	
Male	87 (40.3%)
Female	129 (59.7%)
***Education***	
Illiteracy	0 (0%)
Primary Education	10 (4.6%)
Middle school	20 (9.3%)
High school	60 (27.8%)
Graduate	111 (51.4%)
Postgraduate Education	15 (6.9%)
***Financial data***	
Low income	76 (35.2%)
Medium income	120 (55.6%)
High income	20 (9.3%)

**Fig 1:**
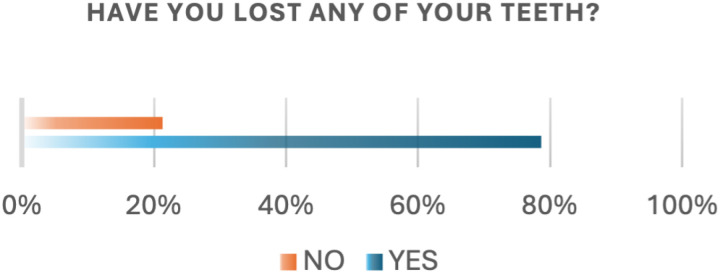
Teeth loss.

The participants’ financial data indicated that 76 (35.2%) had a low income, 120 (55.6%) had a medium income, and 20 (9.3%) had a high income (Table 1). Regarding tooth loss and awareness of its consequences, the study found that 170 (78.7%) of the participants had lost at least one tooth, while 46 (21.3%) had not experienced any tooth loss (Fig 1).

The participants who reported missing teeth were asked a follow-up question regarding the number of teeth lost. Among them, (83%) had lost between and 4 teeth, (12.9%) had lost between 5 and 9 teeth, and (3.9%) had lost between 10 and 13 teeth. Only (0.6%) of participants had lost more than 13 teeth. The number of missing teeth was significantly associated with age (P-value > .0001) and gender (P-value 0.0161; Table 1).

**Fig 2:**
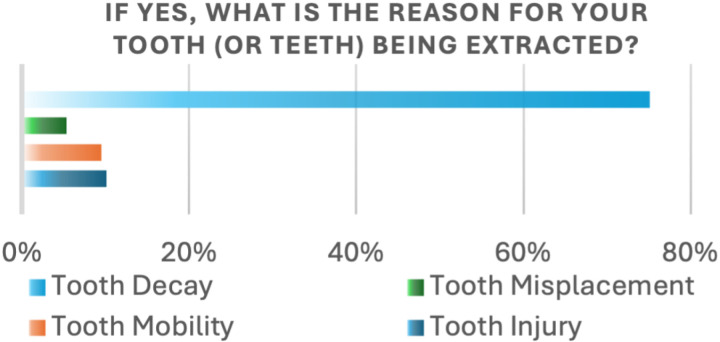
Reasons for extraction.

In another follow-up question, we asked the study participants about the cause of tooth loss. The majority (75.1%) reported dental caries as the primary reason, followed by tooth injury (10.1%), tooth mobility (9.5%), and tooth misplacement (5.3%) (Fig 2).

**Fig 3:**
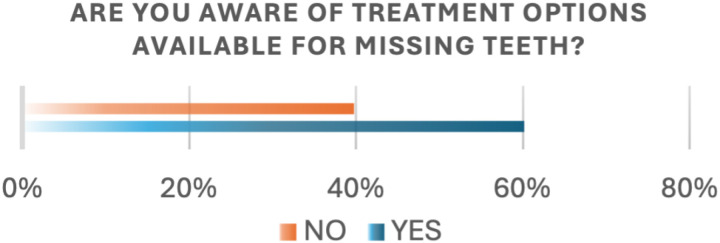
Awareness of treatment options.

Participants were also asked about their awareness of available treatment options, with (60.2%) reporting that they were aware (Fig 3). Lack of awareness regarding available treatment options was significantly associated with age (P-value < .0001), education level (P-value 0.0336), and income level (P-value 0.0037; Table 2). Participants in the age group 18–25 were more likely to report a lack of awareness regarding the available treatment options (OR = 9.00 [1.76–46.09]; p < 0.05). Across education, participants with postgraduate degrees were less likely to report a lack of awareness (OR = 0.08 [1.01–00.64]; p < 0.05). Additionally, Participants with high income were more likely to report a lack of awareness regarding treatment options (OR=4.69[1.43–15.32]; p < 0.05; Table 3).

**Table 2:** Comparison between participants’ demographic variables and number of teeth loss and awareness level.

	Have you lost any of your teeth?		If yes, how many teeth are missing?				If yes, what is the reason for your tooth (or teeth) being extracted?				Are you aware of treatment options available for missing teeth?	Are you aware of the consequences of missing teeth?			If yes, what is the source of your knowledge about the consequences of tooth loss?				
	Yes	No	1-4	5-9	10-13	14+	Tooth Decay	Tooth Mis-placement	Tooth Mobility	Tooth Injury	Yes	No	Yes	No	Dentists	Journals & Books	Social media	Family & Friends	Others
***Age (years)***																			
18-25	27	27	25	2	0	0	15	5	5	2	18	36	25	29	14	5	6	11	6
26-35	61	8	54	0	7	0	46	4	6	3	43	26	46	23	21	5	16	2	112
36-45	34	6	27	2	5	0	24	0	3	7	24	16	23	117	10	2	11	1	6
46-55	40	1	32	11	7	1	37	0	11	3	35	6	34	7	110	1	110	6	7
56-65	7	4	4	0	3	0	5	0	0	2	9	2	6	5	1	1	2	2	2
65+	1	0	0	1	0	0	0	0	1	0	1	0	1	0	1	0	0	0	0
p-value	<.0001		<.0001				0.0006				<.0001	0.0096			**0.4522**				
***Gender***																			
Male	72	15	65	4	3	0	60	5	4	4	47	40	55	32	22	1	13	8	**16**
Female	98	31	77	2	19	1	67	4	12	13	83	46	80	49	35	13	32	4	**17**
p-value	0.2319		0.0161				0.1002				0.1287	0.8579			**0.0111**				
***Education***																			
Primary	10	0	8	0	2	0	10	0	0	0	6	4	5	5	7	0	0	0	**2**
Middle	13	7	14	0	0	0	2	0	4	7	11	9	12	8	6	3	2	2	**3**
High school	54	6	40	5	9	1	43	5	4	3	40	20	45	15	17	4	18	4	**5**
Graduate	79	32	70	1	7	0	64	2	8	3	59	52	60	51	21	6	25	6	**17**
Post-graduate	14	1	10	0	4	0	8	2	0	4	15	1	13	2	6	1	0	0	**6**
p-value	0.0043		0.1284				<.0001				0.0336	0.0186			**0.0337**				
***Income***																			
High	16	4	12	2	2	0	6	2	2	4	16	4	16	4	3	1	1	0	**7**
Medium	93	27	82	2	11	0	76	5	7	6	79	41	79	41	32	13	13	9	**18**
Low	61	15	48	2	9	1	45	2	7	7	35	41	40	36	22	0	0	3	**8**
p-value	0.8896		0.3470				0.0594				0.0037	0.0420			**0.0097**				

**Table 3:** Multivariate analysis for participants’ demographic characteristics and self-reported awareness level and teeth-related issues.

	Are you aware of treatment options available for missing teeth?		Are you aware of the consequences of missing teeth?		Did you have any difficulty in mastication		Did you face any problem in speech?		Did you notice any change in your bite or teeth alignment?		Did you have any TMJ pain or disorder?		Do you think your missing tooth or teeth should be replaced?	
	No %^A^	OR	No %^A^	OR	Yes %^A^	OR	Yes %^A^	OR	Yes %^A^	OR	Yes %^A^	OR	Yes %^A^	OR
***Age***														
** 18-25**	66.7	9.00 (1.76–46.09)^*^	53.7	0.72 (0.1–2.64)	46.3	0.72 (0.2–2.64)	20.4	1.15 (0.22–6.11)	51.9	0.7 (0.21–2.34)	51.9	0.27 (0.07–1.09)	63.0	4.96 (0.62–39.45)
** 26-35**	37.7	2.72 (0.55–13.58)	33.3	1.67 (0.46–6.04)	44.9	0.68 (0.19–2.44)	8.7	0.43 (0.08–2.46)	36.2	1.00 (0.30–3.29)	31.9	0.44 (0.11–1.7)	72.5	3.72 (0.47–29.28)
** 36-45**	40.0	3.00 (0.57–15.74)	42.5	1.13 (0.3–4.32)	60.0	1.25 (0.33–4.8)	15.0	0.8 (0.14–4.62)	30.0	1.51 (0.43–5.28)	45.0	0.32 (0.08–1.3)	77.5	2.85 (0.34–23.89)
** 46-55**	14.63	0.77 (0.13–4.48)	17.1	4.05 (0.96–17.06)	68.3	1.8 (0.46–6.97)	2.4	0.11 (0.01–1.38)	46.3	0.95 (0.27–3.29)	41.5	0.46 (0.11–1.89)	73.1	3.38 (0.41–27.95)
** 56-65**	18.18	1.00	45.5	1.00	54.6	1.00	18.2	1.00	45.5	1.00	27.3	1.00	90.0	1.00
** 65+**	0.00	<.001	0	>.001	100	>999.999	100	>999.999	100	>999.999	0	>999.999	100	<0.001
***Gender***														
** Male**	46.0	1.54 (0.88–2.67)	36.8	0.95 (0.54–1.67)	46.0	0.61 (0.35–1.06)	21.8	4.23 (1.76–10.17)^*^	46.0	1.08 (0.65–1.80)	34.5	0.64 (0.38–1.09)	73.6	1.13 (0.62–2.06)
** Female**	35.7	1.00	37.9	1.00	58.1	1.00	6.2	1.00	38.8	1.00	45	1.00	71.3	1.00
**Education**														
** Primary**	40.0	0.76 (0.20–2.83)	50.0	1.18 (0.32–4.29)	80.0	4.88 (0.99–24.02)^*^	20.0	2.06 (0.39–10.86)	40.0	1.98 (0.58–6.83)	90.0	0.07 (0.01–0.57)^*^	40.0	4.13 (1.22–13.93)^*^
** Middle**	45.0	0.93 (0.36–2.42)	40.0	0.78 (0.3–2.07)	50.0	1.22 (.47–3.16)	20.0	2.06 (0.59–7.19)	30.0	1.82 (0.74–4.50)	30.0	1.3 (0.51–3.28)	70.0	0.98 (0.35–2.73)
** High school**	33.3	0.57 (0.3–1.1)	25.0	0.39 (0.2–0.79)^*^	70.	2.85 (1.46–5.55)^*^	15.0	1.46 (0.58–3.68)	41.7	1.03 (0.57–1.85)	43.3	0.76 (0.42–1.39)	75.0	0.77 (0.38–1.56)
** Graduate**	46.9	1.00	46.0	1.00	45.0	1.00	10.8	1.00	46.0	1.00	38.7	1.00	70.2	1.00
** Post-graduate**	6.8	0.08 (0.01–0.64)^*^	13.3	0.18 (.04–0.84)^*^	66.7	0.61 (0.2–1.90)	0.0	<0.001	26.7	3.13 (1.05–9.31)^*^	26.7	2.44 (0.77–7.77)	100	<0.001
***Income***														
** High**	20.0	4.69 (1.43–15.32)^*^	20.0	0.28 (0.09–0.91)^*^	60.0	0.33 (0.11–1.01)	5.0	0.35 (0.04–2.89)	15.0	3.4 (1.20–9.59)^*^	25.0	3.22 (1.11–9.36)^*^	85.0	0.31 (0.08–1.15)
** Medium**	34.2	2.23 (1.25–4.06)^*^	34.2	0.58 (0.32–1.04)	25.0	1.5 (0.84–2.68)	13.3	1.02 (0.44–2.37)	46.7	0.75 (0.44–1.29)	40.8	1.01 (0.58–1.75)	75.0	0.58 (0.32–1.08)
** Low**	54.0	1.00	47.4	1.00	50.0	1.00	13.2	1.00	40.8	1.00	44.7	1.00	64.5	1.00

^A^Row percentage was used.

* Significant at 0.05.

OR= odd ratio

**Fig 4:**
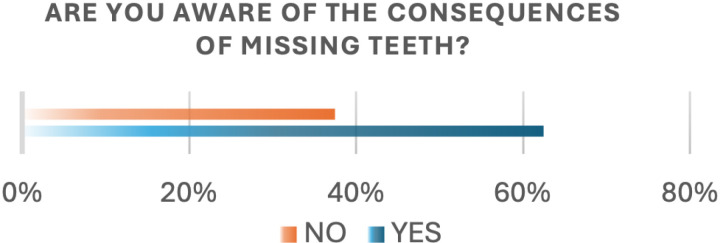
Awareness of tooth loss consequences.

Viewing the awareness of tooth loss consequences, (62.5%) of participants reported being aware of the consequences of tooth loss (Fig 4). However, lack of knowledge about these consequences was significantly associated with age (P-value 0.0096), education level (P-value 0.0186), and income level (P-value 0.0420; Table 2). Participants with school degrees and postgraduate degrees were less likely to report a lack of knowledge about the consequences of missing teeth than those with graduate degrees (OR = 0.39 [0.2–0.79]; p < 0.05) and (OR = 0.18 [0.04–0.84]; p < 0.05). Considering Income level, participants with high income were less likely to report a lack of knowledge compared to others (OR = 0.28 [0.09–0.91]; p < 0.05; Table 3).

**Fig 5:**
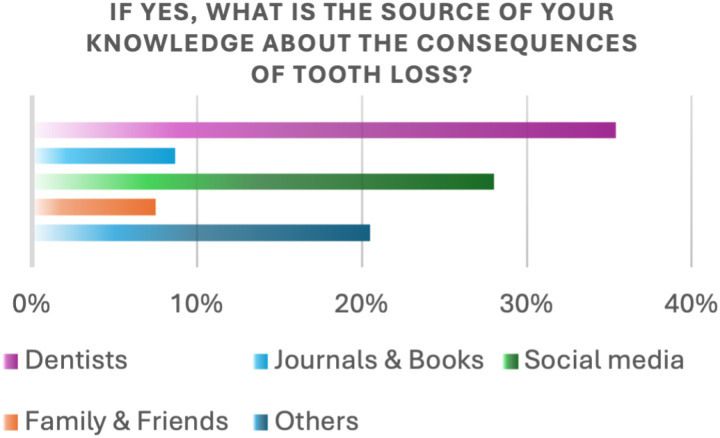
Source of the knowledge about the consequences.

Participants who were aware of the consequences of missing teeth were asked about the source of their knowledge. Dentists were the most frequently cited source (35.4%), followed by social media (28%), family and friends (7.5%), and journals and scientific books (8.7%) (Fig 5).

**Fig 6:**
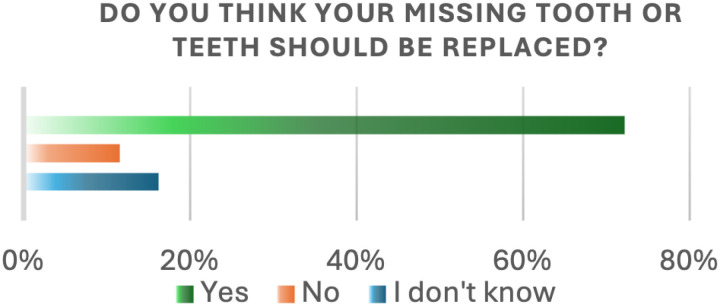
Missing teeth should/shouldn’t be replaced.

Regarding the impact of tooth loss, (53.2%) of participants reported difficulty during mastication, (12.5%) experienced speech problems, and (41.7%) noticed changes in their bite or teeth alignment. Additionally, (40.7%) of participants reported experiencing temporomandibular joint (TMJ) pain or any TMJ-diagnosed disorder. When asked if they believe their missing teeth should be replaced, (72.2%) responded “yes,” (11.6%) responded “no,” and (16.2%) were unsure (Fig 6). There was a significant relationship between age and gender and speech problems due to missing teeth (P-value = 0.0091), and (P-value = 0.0007), respectively. Male participants were more likely to report speech difficulties than females (OR = 4.23 [1.76–10.17]; p < 0.05; Table 3). Furthermore, TMJ pain or disorder relayed to missing teeth was significantly associated with age (P-value 0.0305; Table 4).

**Table 4:** Comparison between participants’ demographic variables and teeth-related issues.

	Did you have any difficulty in mastication		Did you face any problem in speech?		Did you notice any change in your bite or teeth alignment?			Did you have any TMJ pain or disorder?		
	Yes	No	Yes	No	Yes	No	I Don’t Know	Yes	No	I Don’t Know
***Age (years)***										
18-25	25	29	11	43	28	19	7	28	23	**3**
26-35	31	38	6	63	25	25	19	22	32	**15**
36-45	24	16	6	34	12	20	8	18	17	**5**
46-55	28	13	1	40	19	18	4	17	23	**1**
56-65	6	5	2	9	5	5	1	3	8	**0**
65+	1	0	1	0	1	0	0	0	11	**0**
p-value	0.1398		0.0091		0.2285			**0.0305**		
***Gender***										
Male	40	47	19	68	40	28	19	30	45	**12**
Female	75	54	8	121	50	59	20	58	59	**12**
p-value			0.0007		0.1241			**0.2553**		
***Education***										
Primary	8	2	2	8	4	6	0	9	1	**0**
Middle	10	10	4	16	6	10	4	6	11	**3**
High school	42	18	9	51	25	20	15	26	25	**9**
Graduate	50	61	12	99	51	41	19	43	56	**12**
Post-graduate	10	10	0	15	4	10	1	4	11	**0**
p-value			0.3647		0.1703			**0.0283**		
***Income***										
High	5	15	1	19	3	14	3	5	15	**0**
Medium	72	48	16	104	56	41	23	49	53	**18**
Low	38	38	10	66	31	32	13	34	36	**6**
p-value	0.0115									

**Fig 7:**
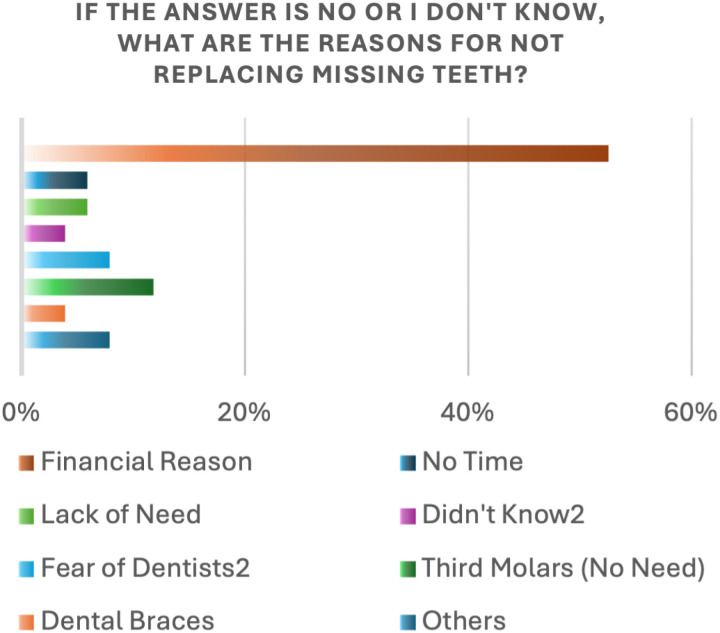
Reasons for not replacing missing teeth.

Participants were asked about the reasons for not replacing their missing teeth. The most reported reason was financial costs by (52.6%) of respondents. This was followed by the lack of perceived need to replace the third molars (11.8%), fear of dentists (7.9%), and lack of time (5.9%) (Fig 7). Regarding participants’ awareness of teeth-related problems, knowledge of tooth consequences such as tooth drifting and jaw atrophy was significantly associated with gender and education levels (Table 5).

**Table 5:** Comparison between participants’ demographic variables and awareness regarding teeth problems and other teeth-related issues.

	Do you think your missing tooth or teeth should be replaced?			If the answer is no or I don’t know, what are the reasons for not replacing missing teeth?								Did you ever know that losing a tooth cause tooth drifting?		Did you ever know that losing a tooth causes atrophy of the jaw bones?	
	Yes	No	I Don’t Know	Financial Reason	No Time	Lack Of Need	Didn’t Know	Fear of Dentists	Third Molars (No Need)	Dental Braces	Others	I Know	I Don’t Know	I Know	I Don’t Know
***Age (years)***															
18-25	34	5	15	15	0	5	1	3	4	4	5	23	31	23	**31**
26-35	50	10	9	25	4	0	2	5	5	5	5	28	41	35	**34**
36-45	31	5	4	15	4	0	3	3	7	7	0	24	16	19	**21**
46-55	30	4	7	20	1	3	0	1	2	2	2	20	21	15	**26**
56-65	10	1	0	4	0	1	0	0	0	0	0	5	6	2	**9**
65+	1	0	0	1	0	0	0	0	0	0	0	0	1	0	**1**
p-value	0.4127			0.2440								0.3928		**0.2891**	
***Gender***															
Male	64	10	13	33	0	4	2	6	9	2	1	30	57	30	**65**
Female	92	15	22	47	9	5	4	6	9	4	11	70	59	64	**57**
p-value	0.9139			0.0850								0.0042		**0.0278**	
***Education***															
Primary	4	4	2	7	2	0	0	0	1	0	0	7	3	8	**2**
Middle	14	2	4	5	0	0	0	3	0	3	5	12	8	10	**10**
High school	45	5	10	2	1	0	0	2	8	2	2	24	36	19	**41**
Graduate	78	14	19	3	3	4	5	9	9	4	3	46	65	45	**66**
Post-graduate	15	0	0	2	3	2	1	0	0	2	2	11	4	12	**3**
p-value	0.0527			<.0001								0.0374		**0.0014**	
***Income***															
High	17	1	2	4	1	1	0	1	0	2	0	16	4	12	**8**
Medium	90	11	19	43	3	5	4	6	14	3	9	56	64	54	**66**
Low	49	13	14	33	5	3	2	5	4	1	3	28	48	28	**48**
p-value	0.2593			0.2216								0.0026		**0.1576**	

## Discussion

Regarding awareness of the consequences of missing teeth, the study revealed that a significant portion of the participants were unaware of its effect. Specifically, 53.7% of the participants were unaware that tooth loss can lead to the drifting of adjacent teeth, while 56.5% did not know that tooth loss can result in jawbone atrophy. Furthermore, there was a significant relationship between a lack of knowledge regarding available treatment options and factors such as age, education, and income level. The purpose of this study is to investigate the level of understanding and awareness among adults in Qassim regarding the challenges associated with tooth loss and the various treatment options available.

In our study, approximately 78.7% of participants had experienced tooth loss, which is significantly higher than the 49.4% reported by Suganna et al. This difference may be attributed to their study including participants from five different regions in Saudi Arabia, while our study focused solely on the Qassim population. Concerning awareness of treatment options, 60.2% of the study participants were informed, which was lower than the 87% reported by Alshehri et al and Hussain et al. [16,17]. This discrepancy could be due to differences in the age distribution between our study and theirs. In particular, younger individuals were found to have a greater awareness of treatment modalities and complications, as indicated by a study by Abdulrahman et al. [18].

Regarding participant awareness of the consequences of tooth loss, our study indicates that 62.5% of participants are aware of these consequences, which is higher than the findings of Alshehri et al. This difference could be explained by the higher illiteracy rates observed in Alshehri et al.‘s study [16].

In our study, 35.4% of participants reported obtaining information about the effects of tooth loss from dentists, while 28% learned from social media. These findings align with those of Mously et al. where 43.3% cited social media and 53.4% cited dentists as their primary sources of information [19].

Approximately 53.2% of participants in our study reported having trouble with masticating, 12.5% reported having speech problems, 41,7% noticed changes in their bite or teeth alignment, and 40.7% reported pain or disorders related to their TMJ. Compared to Dosumu et al., mastication difficulties were more common in our study, as their research reported 40.9% of participants experienced this issue [20]. Speech difficulties were more prevalent in our study than in Alshehri et al. study, which found that 19.5% of participants had trouble speaking. The prevalence of TMJ pain and disorder in our study closely mirrors Alshehri et al.’s finding of 38% [16]. Additionally, changes in bite and tooth alignment were reported more frequently in our study than in Dosumu et al.’s study, where the prevalence was 30.1% [20].

In our study, 72.2% of the participants stated that they were aware of the importance of replacing missing teeth, which is lower than the percentage reported by Alshehri et al. [16]. The cost was cited by 52.6% of participants in our study as the main barrier to replacing lost teeth, which aligns with findings from Suganna et al. [17]. Their study found that, 68.1% of participants identified cost as the primary restraint to replacing missing teeth.

In contrast to the findings of Abdulrahman et al. [18], which demonstrated a higher knowledge of awareness of treatment modalities among younger populations, our statistical analysis using the chi-square test revealed a significant relationship between age and lack of knowledge regarding available treatment options, particularly in young age groups. The awareness of and approach to managing tooth loss are significantly correlated with age, income, and educational level.

There are several limitations to this study. First, an online survey was used to collect data, which may introduce recall biases, whereas interviews and clinical oral examinations would be more effective in reducing these biases. Second, self-selection biases might have occurred due to the small sample size and the voluntary nature of participation. Lastly, this study focused solely on the Qassim population. Future research should aim to include a larger sample size and encompass the broader Saudi population.

## Conclusion

In our study, a significant number of participants were unaware of the consequences of replacing missing teeth and the available treatment options. Younger individuals demonstrated lower awareness of these treatment options compared to other age groups. However, participants with higher incomes were less likely to report a lack of knowledge, and those with postgraduate degrees had greater awareness compared to other groups.

These findings highlight the critical need to educate all individuals about their oral health and tooth loss while considering their different socioeconomic conditions. Moreover, promoting treatment options tailored to each patient’s needs can encourage them to seek care, improve treatment outcomes, and enhance overall satisfaction.

## Supporting information

S1 File DATA_Assessment of awareness level english. (CSV)

## Abbreviations

QoL: quality of life
TMDs: temporomandibular joint disorder
TMJ: temporomandibular joint.
